# Some Account of the Tanjore Antidotes for the Bite of a Mad Dog; and Also for the Bite of Venomous Serpents

**Published:** 1789

**Authors:** 


V. *Some Account of the Tanjore Antidotes for the Bite of a mad Dog; and alſo for the Bite of venomous Serpents.*

HAVING been favoured with copies of ſeveral papers from the Eaſt Indies relative to ſome ſuppoſed antidotes for the bite of mad dogs, and of venomous ſerpents, which have been deemed of ſufficient importance to

 merit merit the attention of Government at Madraſs, we think it right to give ſome account of them in this work.

The papers in queſtion conſiſt of copies of letters from the Secretary of the Governor and Council at Madraſs, addreſſed to Dr. James Anderſon, Phyſician General, and to the other members of the Hoſpital Board at that place, and dated in September and October, 1788; together with extracts from the minutes of the Hoſpital Board, dated in September and November, 1788; and directions for preparing and adminiſtering the remedy for the bite of a mad dog, and likewiſe the ſnake pills.

From theſe papers we learn that his Excellency the Governor of Madraſs, having been informed that Samoo-Vell, a native of Tanjore, was in poſſeſſion of a compoſition which had the reputation of being efficacious in the prevention of hydrophobia, and of another called ſnake pills, with which he was ſaid to cure perſons bit by venomous ſerpents, very humanely directed him to be ſent to Madraſs for the purpoſe of having the merits of theſe remedies aſcertained by the members of the Hoſpital Board.

It

It appears that the principal and indeed the only active ingredient of the remedy for the prevention of hydrophobia is the dried leaf of the Datura Stramonium, or thorn apple, given three days ſucceſſively in a doſe of three drachms and a half, which it ſeems is ſufficient in a few hours to excite intoxication. About three hours after its exhibition the patient is directed to drink liberally of a decoction of rice; and at night cold water is to be poured on his head. On the fourth and fifth days he is to obſerve a ſtrict regimen, and on the ſixth is to return to his uſual mode of living. — No particular inſtances are related in which this remedy has been tried; and indeed as a prophylactic in theſe melancholy caſes it has, we fear, no better claim to our confidence than the internal remedies employed on ſimilar occaſions in Europe.

In the minutes of the Hoſpital Board we find Dr. Anderſon, the Phyſician General, very prudently pointing out the dangerous properties of the thorn apple, the leaves of which, he obſerves, are, in India, commonly rubbed on the inſide of the ſtill and pots for toddy, to render that liquor more intoxicating; and "the ſeeds "of one apple," he adds, "are ſufficient for

"thoſe

" those who perpetrate self murder, (which is " too frequently the consequence of family dis- " sensions in India) to induce immediate apo- " plexy, and death in five or six hours."

The other remedy, or snake pill, as it is called, is composed of

" The roots of Velli-navi,

" and Neri-visham;

" the kernels of Ner-valum;

" white Arsenic;

" Pepper;

" and quicksilver; of each equal parts."

Of the first three of these ingredients, which are procured from the Malabar Coast, Samoo Vell could give no satisfactory account; but it seems that the roots of Velli-navi and Neri-visham are poisonous, and that the kernel of the Ner-valum is a violent drastic purgative.

In preparing these pills the quicksilver is directed to be rubbed with the juice of the *Asclepias Gigantea* Linn. till the globules are extinguished. The arsenic (previously levigated) and the other ingredients, reduced to a powder, are then to be added, and the whole beat up with the juice just now mentioned to a consistence fit to be divided into pills, which are to weigh about six grains each.

After

After dilating the wound, one, and in ſome caſes two, of theſe pills are directed to be given, according to the urgency of the ſymptoms, and repeated at the end of a quarter or half an hour if the ſymptoms do not then begin to ſubſide.

From the minutes of the Hoſpital Board it would ſeem that Mr. Duffin, a member of that Board, and Head Surgeon at the Preſidency of Madraſs, entertains a very favourable opinion of this medicine, the formula of which he procured through the medium of the Reverend Mr. Swartz, a Daniſh miſſionary, during his reſidence at Vellore.

The firſt trial he made of it was in the caſe of a Malabar woman, who had been bit in the leg by a Cobra de Capella, or hooded ſnake, and whoſe recovery Mr. Duffin aſcribes to the effect of this medicine; but as in this caſe it was not adminiſtered till ten hours after the bite, and eight hours more elapſed before the ſymptoms began to abate, it may perhaps be doubted whether the recovery was not owing to the inſufficiency of the poiſon to deſtroy life, rather than to any ſpecific operation of the remedy employed to counteract it.

Of the viper, it has been obſerved by Abbé Fontana, that, from the quantity of its virus requiſite quisite to produce death in different animals, it is extremely doubtful whether it ever proves fatal to the human species; and the same celebrated writer has collected a few instances, from authors, which render it probable that even the bite of a rattle snake is not always fatal to man *.

The subject of Mr. Duffin's second trial of this medicine was a soldier, who had been bit in one of his fingers by a small brown snake, and had applied a ligature around the finger above the wound. One pill was administered about a quarter of an hour after the bite; and, upon the ligature's being then loosened, the patient complained of a violent pain that extended upwards to his shoulder.

At the end of three hours, as the pain did not abate, and the medicine had produced no sensible effect, a second pill was administered. The symptoms were now more distressing, and the patient became delirious and was affected with spasms.

In this alarming state a third pill was given; this was succeeded by a violent vomiting and

* See his *Traité sur le Venin de la Vipere*, Part III.; and the fifth volume of this Journal, pages 13 and 14.

purging; purging; and the next morning, when Mr. Duffin ſaw the patient, he was free from pain, and complained only of great weakneſs. On each of the two following days he took one pill, and on the fourth day was able to return to his duty.

One circumſtance, which ſeems to be a material one, muſt not be omitted to be mentioned, and that is the local treatment; for the wound, it ſeems, both in this and the preceding caſe, was freely dilated, and dreſſed with mercurial ointment.

Since theſe two caſes upwards of fifty others, of perſons bit by different kinds of ſnakes, have occurred, to Mr. Duffin, in which he has adminiſtered theſe pills; but as none of theſe caſes were attended with dangerous ſymptoms, he thinks it probable that many of the ſnakes were not venomous. The pills, he obſerves, generally occaſioned a nauſea and purging; but ſeldom operated with violence.

This, however, does not accord with the ſentiments of another member of the Hoſpital Board, Dr. Anderſon, on this ſubject, who declares he has been "credibly informed that the "exhibition of the ſnake pills has been attended "with burning pain of the ſtomach and

 "bowels,

" bowels, violent vomiting, purging, and, in " some cafes, even with death, as might be ex- " pected from the arfenic they contain."

Dr. Anderfon farther obferves, that two thirds of the fnakes in the Eaft Indies are not venomous*; and " if thefe pills," fays he, " are " adminiftered to all who are bit, three perfons " muft run the rifk of their lives under the idea " of faving one by a dofe of arfenic."

" If the part," he adds, " is cut out within " a quarter of an hour after the bite of our " moft venomous fnakes, and a ligature is " made above the wound, no ill confequence " enfues; and even when this has been neg- " lected, and giddinefs, the firft morbid fymp- " tom, has occurred, the life of the patient has " been faved by fupporting the vis vitæ with

* Of one hundred and thirty-one fpecies of ferpents enumerated by Linnæus, in his Syftema Naturæ, only twenty-three are marked as venomous, which is not much more than one in fix; and Dr. Gray, it feems, has examined one hundred and fifty-four fpecies of ferpents, of which number only twenty-fix appeared to be venomous.—See an ingenious paper on this fubject, by the latter, in the Philofophical Tranfactions, Vol. LXXIX. Part I. entitled " Obfervations on the " Clafs of Animals, called, by Linnæus, Amphibia; particularly on the means of diftinguifhing thofe ferpents which " are venomous from thofe which are not fo."—Editor.

" wine

"wine and opium." — This agrees with what we find mentioned in a late publication by Lieutenant Paterſon, who, during his reſidence in India, became acquainted with this medicine, but who, in a caſe in which it could not be procured, ſaw brandy, and Madeira wine, given ſo as to intoxicate the patient, prove equally efficacious *.

The ſnake pills have likewiſe been recommended againſt the bite of a mad dog; and it appears that Mr. Duffin, while ſurgeon to the garriſon at Veliore, adminiſtered them to three ſepoys and eight children who were bit by mad dogs in October, 1787, and that all of theſe perſons eſcaped the infection, while three other children,

* "The ſouthern countries of Hindoſtan abound with the "ſmall ſnake, called the Covra Manilla, which is well "known to be very poiſonous. The Bramins tell us, that "they can adminiſter complete relief in the moſt deſperate "caſes; but their mode of practice has hitherto been kept a "ſecret from Europeans.—Colonel Fullarton, however, pro-"cured a ſmall box of theſe pills from the Reverend Mr. "Swartz, a miſſionary at Tanjore; and at the ſiege of Car-"rore we had an opportunity of proving the effects of them. "One of our ſepoys was bitten, and ſo ill that we deſpaired "of his life. The Colonel gave him one of the pills, which "ſeemed to act as a very ſtrong opiate for ſome time, and

 "threw children, who made ufe neither of this nor of any other means of prevention, died of hydrophobia. Thefe facts are afcertained by the refpectable teftimony of Mr. Duffin himfelf, who had the care of the patients, and likewife by the following certificates from Colonel Nixon, Commandant, and other gentlemen of the garrifon at Vellore:

" I atteft, that in October laft, when the fe-
" poys were bit, I had a report that feveral
" children were bitten, whom I directed to be
" taken to Dr. Duffin to adminifter to them the
" Tanjore medicine. I underftand they are
" all well at this period, except one who died a
" natural death two months after the bite. I

" threw him into a delirium; in two days, however, the man
" was perfectly recovered.
" We had alfo a fecond proof of their utility, though the
" man did not appear to be fo ill as on the former occafion.
" I was witnefs to a third cafe, where we could not procure
" thefe pills. A fervant of Lieutenant Smith, in the fame
" regiment with myfelf, was bitten. The Lieutenant gave
" him nothing but brandy and hot Madeira wine, and kept
" him in a ftate of intoxication for twenty-four hours; the
" next day the pain was gone, but the man continued indif-
" pofed for fome time." — See a Narrative of four Journies into the Country of the Hottentots and Caffraria; by Lieut. William Paterfon. 4to. London, 1789. p. 166.

" have alſo an account that three children were " bit, who did not receive the medicine, and, " from the parents relation, there is every rea- " ſon to believe they died with ſymptoms of " canine madneſs in the ſpace of a month after " the bite.

" Eccles Nixon, Col. Commandt.
" Vellore, March 14, 1788."

" Liſt of the Men of the 21ſt Battalion of " native Infantry, bit by mad Dogs in Oc- " tober, 1787.

| " *Mens Names.* | *Rank.* | *Company.* | *Age.* | *Where wounded.* |
|---|---|---|---|---|
| " Vencata Ramah, | Sepoy, | 4th. | 24 | Very much torn in the wriſt and arm. |
| " Papa Naick, – | Do. | 6th. | 45 | Bit in the lower part of the thigh. |
| " Raſapan, – – | Do. | 2d Grenad. | 28 | Bit in the leg. |

" We atteſt the above-mentioned men were " bit by mad dogs in October laſt. They re- " ceived medicines from Dr. Duffin, and are " well at this period. The dog which bit Ven- " cata Ramah bit a dog of the Fort Adjutant's " that went mad ten days after.

" W. VIGORS,

" Captain commanding the 21ſt Batt. Nat. Inf.

" JOHN TAYLOR, Lieutenant and Adjutant.
" March 14, 1788."

It

It appears from Mr. Duffin's account that in all of thefe cafes the wound was largely dilated and dreffed with mercurial ointment; fo that the facts in queftion may, perhaps, be thought to prove as much in favour of this part of the treatment as they do of the pills. At any rate, however, it muft be confidered as a very curious circumftance, that eleven perfons, who were treated in one particular way, fhould all of them be free from infection at the end of five months, while three others, to whom nothing was done, died of hydrophobia within a month after the bite; but to fhow how much caution is neceffary in the admiffion and application of facts of this kind, it may not be improper to remind our readers, that when the lichen cinereus terreftris was firft introduced to the notice of the public, as a fuppofed remedy againft the hydrophobia, it was faid, by Sir Robert Moray, to have been "given to a whole kennel of dogs "bitten by a mad one, which were all cured "except one of them, to whom none of it was "given*."

Government, defirous of having the effects of thefe compofitions more fully afcertained,

* Birch's Hiftory of the Royal Society, Vol. II. page 492.

have, have, it ſeems, given orders that a quantity of each of them be prepared and ſent to the ſurgeon of every ſtation in the preſidency of Madraſs; with directions to diſtribute them, gratis, to all who may require them, and to tranſmit an account of their effects to the Hoſpital Board; ſo that, at ſome future time, the public may, perhaps, receive farther and more ſatisfactory information on this ſubject.

