# Errata

**Published:** 1789

**Authors:** 


					294> 1. 8; for eleven perjons, read ten ptrjonsx

				

